# An Indo-Pacific humpback dolphin (*Sousa chinensis*) severely injured by vessel collision: live rescue at sea, clinical care, and postmortem examination using a virtopsy-integrated approach

**DOI:** 10.1186/s12917-022-03511-1

**Published:** 2022-11-26

**Authors:** Brian Chin Wing Kot, Heysen Hei Nam Ho, Paolo Martelli, Sarah M. Churgin, Nimal Fernando, Foo Khong Lee, Henry Chun Lok Tsui, Tabris Yik To Chung

**Affiliations:** 1 Department of Infectious Diseases and Public Health, Jockey Club College of Veterinary Medicine and Life Sciences, City University of Hong Kong, Kowloon Tong, Hong Kong; 2 Centre for Applied One Health Research and Policy Advice, City University of Hong Kong, Kowloon Tong, Hong Kong; 3 Veterinary Hospital, Zoological Operations & Conservation, Ocean Park Corporation, Aberdeen, Hong Kong; 4 Laboratory Animal Services Centre, The Chinese University of Hong Kong, Shatin, Hong Kong

**Keywords:** Cetacean, Vessel collision, Indo-Pacific humpback dolphin *Sousa chinensis*, Rescue, Rehabilitation, Virtopsy, Computed tomography, Magnetic resonance imaging

## Abstract

**Background:**

Vessel collision induces blunt and sharp force traumas to aquatic animals and is a leading anthropogenic impact affecting cetaceans worldwide. Vessel collision is an important threat affecting vulnerable coastal cetaceans such as the Indo-Pacific humpback dolphins (*Sousa chinensis*) which reside in coastal waters of Hong Kong amongst heavy marine traffic.

**Case presentation:**

A severely injured subadult *S. chinensis* was sighted in the waters off southwestern Hong Kong with four gaping incision wounds on its dorsum. It was in poor body condition and seemed unable to use the fluke effectively. The deepest wound located at the caudal peduncle near the base of the fluke and exposed the underlying fractured caudal vertebrae. The dolphin was monitored in the field over three weeks and eventually captured for medical intervention as veterinary assessment indicated progressive and life-threatening deterioration. During rehabilitation, the dolphin demonstrated initial signs of improvement over the first 36 hours as supported by diagnostic tests but then deteriorated rapidly. It was humanely euthanised after three days of rehabilitation. Postmortem investigation was carried out using virtopsy (postmortem computed tomography and magnetic resonance imaging) and conventional necropsy, with special attention to the traumatic musculoskeletal injuries caused by vessel collision and also revealed acute gastrointestinal compromise and respiratory disease that further hampered the rehabilitation.

**Conclusion:**

In cetaceans, the prognosis for recovery from injuries caused by vessel collision depends on the extent, location, and gravity of the injuries (i.e., superficial, deep, penetrating, blunt vs. sharp, fresh vs. septic), as well as the health status of the individual and its ability to respond to the insult. Injuries extending deep into the vertebral column may lead to delayed death and associated welfare issues. The prognosis of this case was likely poor given the severity and location of the injuries, but the attempted rehabilitation and postmortem investigation provided valuable insights for clinical management if similar cases are encountered in the future. Being able to non-invasively assess and document traumatic injuries and other pathologies, diagnostic imaging is particularly useful in the clinical assessment and postmortem investigation (virtopsy) of cases with vessel-induced injuries.

## Background

Vessel collisions cause injuries and mortalities in marine animals worldwide and have been documented to affect at least 48 species of cetaceans including both mysticetes and odontocetes [1, 2]. Traumatic injuries from vessel collision can be classified into two types: (1) blunt force trauma from colliding with the non-rotating parts of the vessel such as the bow or the hull; this commonly features focal haemorrhages, organ and muscle disruption, and skeletal fractures [3, 4], and (2) sharp force trauma induced by the rotating propellers which typically presents as a single or a series of linear to curvilinear chop wounds and in some cases, amputation [4–6]. Blunt and sharp force traumas can also co-occur in vessel collision events. The severity and outcome of injuries from vessel collision incidents vary, ranging from immediately fatal, causing prolonged debilitation prior to eventual death, to inflicting sublethal but irreversible injuries that may compromise long-term fitness and survival, and sometimes apparent full recovery [2, 3, 7, 8]. Vessel collision also constitutes as a welfare issue for the affected animals, especially in cases with delayed mortality after substantial injuries and prolonged debilitation [9, 10].

Small cetaceans occupying coastal waters near urbanised centres are particularly impacted by anthropogenic impacts, especially from collision with small- and medium-sized vessels [1]. In Hong Kong and the Pearl River Estuary region, vessel collision poses a severe threat to the survival of the vulnerable residential Indo-Pacific humpback dolphin *Sousa chinensis* (SC) [11, 12], which has a rapidly declining population over the past two decades [13–15]. Coastal waters in Hong Kong have heavy maritime traffic related to various human activities, including coastal construction projects, passenger and cargo transportation, fishery operations, and dolphin-watching tourism [16, 17]. Jefferson et al. [18] reported that traumatic injuries from vessel collision were observed in 4.5% of stranded SC in Hong Kong between 1995 and 2004. However, stranding data from 2017 to 2020 identified vessel collision as the primary cause of mortality in 21% of all stranded cetaceans in Hong Kong waters, including both SC and another residential species, the Indo-Pacific finless porpoises (*Neophocaena phocaenoides*) [11], which raises concerns for the conservation and continuous survival of cetaceans in Hong Kong waters. In addition, evidence of sublethal injuries attributed to propeller strike was observed in 1.2–1.8% of free-swimming SC sighted during photo-identification surveys between 1995 and 2004 [18]. Thus, vessel collision is recognised as one of the main threats to the long-term survival of SC and Indo-Pacific finless porpoise populations in Hong Kong waters [2, 11].

Previous reports documenting cetacean injuries due to vessel collision events have provided insights on the injury progressions and outcomes (e.g., [7, 8, 19–23]). These reports enabled better decision making and clinical management when such events are unfortunately encountered. Herein we report the case of a severely injured SC from Hong Kong waters suffering from multiple sharp traumas likely caused by propeller strike, with thorough documentation on the field monitoring, rescue, clinical care, and postmortem examination using a virtopsy-integrated approach.

## Case presentation

### Field monitoring and rescue

**Fig. 1:**
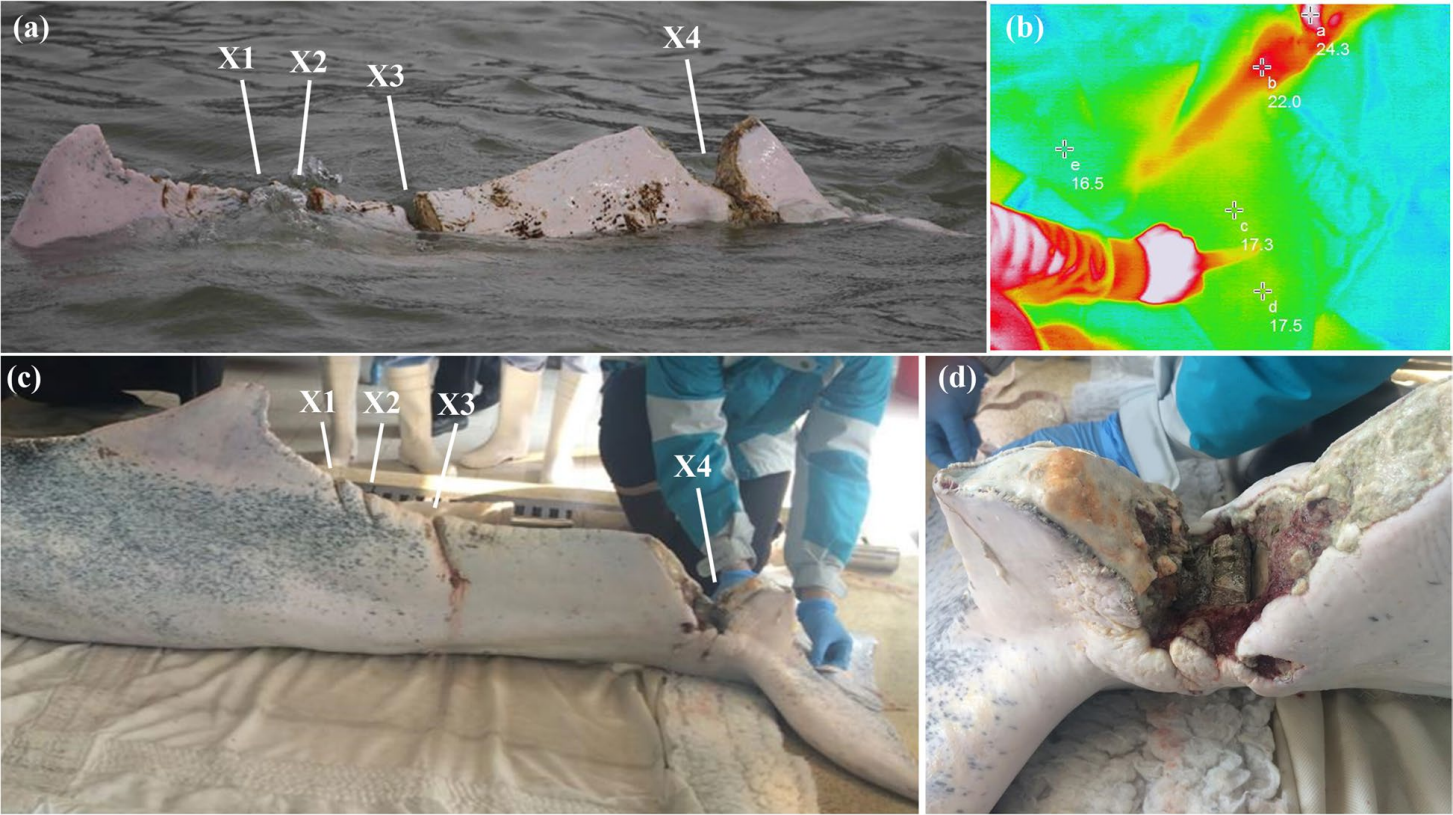
**a** Field photograph of WL212 (also known as “Hope” or SC15-16/01), a severely injured Indo-Pacific humpback dolphin (*Sousa chinensis*) from Hong Kong waters, showing four incision wounds on the dorsum of lumbocaudal region (X1-X4) likely caused by propeller strike, with X4 being the most severe with suspected underlying vertebral fracture. **b** On-site thermography imaging after rescue revealed that the blood supplies to the fluke were still intact (indicated by points c, d, e) despite the severe injury at the caudal peduncle (X4). **c** Photograph of WL212 following euthanasia (taken three weeks after monitoring and three days after rescue), showing the four incision wounds on the dorsum. **d** Close-up of X4 following euthanasia. The wound fractured and exposed the underlying vertebrae. Soft tissues around the wound were macerated and infected with granulation tissues at the periphery

The severely injured SC, named “Hope” by the media in Hong Kong and hereafter referred to as WL212 (also known as SC15-16/01 in the Hong Kong cetacean stranding records managed by the Agriculture, Fisheries and Conservation Department of the Hong Kong Special Administrative Region Government), was first sighted in coastal waters near Tai O, Lantau Island, Hong Kong (22º15’N, 113º50’E) on 16^th^ January 2015 by local cetacean researchers. Through established photo-identification catalogues, the individual was matched to a known individual (WL212) that had been repeatedly sighted in Hong Kong waters since 2012 [24]. Based on the body size and colour pattern, WL212 was classified as a subadult [25] (or as a “speckled” individual between juvenile and adult [26, 27]). Externally, WL212 suffered from four deep lacerations to the dorsal aspect of the caudal region (X1-X4; Fig. 1a), and the deepest wound was at the caudal peduncle with suspected underlying vertebral fractures. Continuous field monitoring of WL212 was carried out via collaboration between the Agriculture, Fisheries and Conservation Department of the Hong Kong Special Administrative Region Government, Ocean Park Conservation Foundation Hong Kong, and local cetacean research groups.

The dolphin was unable to use its fluke effectively and mostly dragged the tail fluke behind. Despite of this, it was able to evade approaching vessels by paddling with its pectoral flippers. Monitoring of the dolphin was also hindered by weather conditions and the limited visibility of the waters. However, it was observed that the body condition of WL212 deteriorated over a short time, and it became emaciated. The wounds gaped more and the soft tissues around X4 appeared more macerated and infected. WL212 started to display begging behaviours and began to accept fish offered by local fishermen and the rescue team. Over the three weeks of field monitoring, multiple rescue attempts were unsuccessful due to adverse weather, lack of expertise in dolphin capture, inadequate equipment, and logistical difficulties. On the third week, oral sedation using 80 mg diazepam (0.6 mg/kg) and 50 mg butorphanol (0.37 mg/kg) was mixed into fish and offered to WL212 during the sixth rescue attempt [28]. The dolphin was in a suitable location and sufficiently sedated three hours later and was captured from a vessel using a hoop net and swimmers. It was then placed in a stretcher and lifted onto the stern platform of the vessel for on-site assessment.

### On-site assessment and decision-making

Immediate assessment of WL212 revealed that dolphin had four lacerations on the dorsal aspect and was severely emaciated (cachexia) because of the traumatic injuries and sepsis. In particular, the peduncle wound (X4) was the most severe, appeared gangrenous, and was foul smelling with exposed vertebrae that were also necrotic. Hence, the possibility of recovery after one-off medical treatment and immediate release was deemed impossible. Despite the severity of the peduncle wound, thermography and venipunctures revealed that the blood supply to the tail fluke was still present (Fig. 1b); therefore, there was no indications for immediate euthanasia. As the dolphin was unsuitable for immediate release, the attending veterinarians decided to transport WL212 back to medical facilities for further veterinary assessment, supportive medical care, and rehabilitation.

### Veterinary interventions

**Fig. 2:**
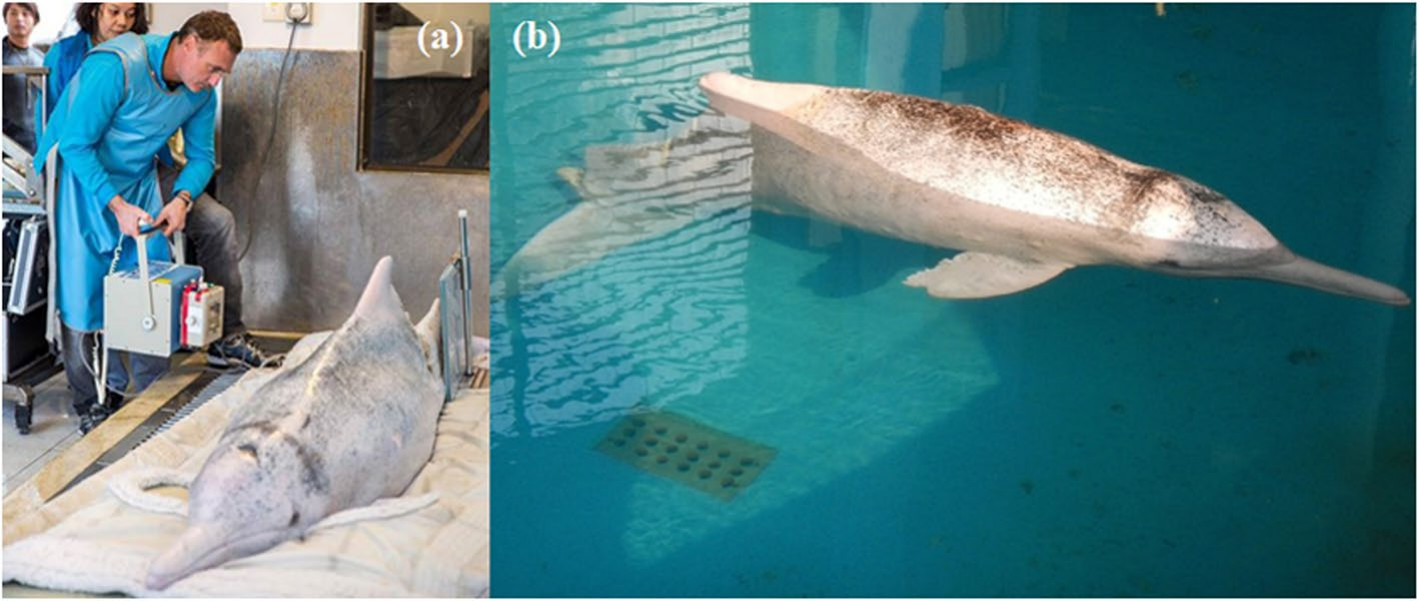
Photographs of the severely injured *Sousa chinensis* (WL212) during clinical care and rehabilitation. **a** Poolside radiography of the peduncle wound (X4) being performed by a certified veterinarian. **b** WL212 in the pool of the rehabilitation facility, notably showing X4 at the caudal peduncle

Upon admission, antibiotic and analgesic medications were administered to WL212, including intramuscular injection of carprofen (2 mg/kg, Rimadyl®, Zoetis, MI, USA), cefovecin (8 mg/kg, Convenia®, Zoetis, MI, USA), and diazepam (0.22 mg/kg, ilium Diazepam®, Troy Animal Healthcare, Australia), and intravenous injection of ceftazidime (30 mg/kg, Sandoz®, Sandoz, Australia). The dolphin was also tube fed with 2.5 L of fluids and fish mash for rehydration and sustenance. Poolside radiography (Fig. 2a) revealed that several vertebral bodies at the peduncle wound were fractured and luxated, while all surrounding intervertebral disc spaces were collapsed. In addition, the spinous process of a caudal vertebra at X3 was also fractured. Consistent with the earlier thermography assessment, blood supply to the tail fluke was still present and it was possible to sample blood from all four major fluke vessels. Intravenous antibiotics were administered in the dorsal fin to avoid accidentally damaging the fluke vessels (phlebitis). Preliminary blood analysis showed a moderate leucocytosis (13,460 cells/µL), elevated fibrinogen (665 mg/dL), mildly elevated urea (22.1 mmol/L), and hyperglycaemia (10.2 mmol/L) based on comparisons to other cetacean species as there are no published reference ranges for SC. On gross examination, the dolphin had a near absence of blubber, chronic muscle wastage, and was severely underweight (135 kg) relative to its body size (233 cm). The prognosis for the animal was poor considering the severe traumatic injuries, chronic sepsis, and emaciation. The animal was placed in a pool with water 8 °C warmer than the winter waters (~ 14 °C) and with access to a warm water pipe delivering water at 28–30 °C (Fig. 2b).

Following initial medical treatment, WL212 was stable but still in critical condition. The demeanour and strength of the dolphin improved with hydration and warmer water. The soft tissues around the peduncle wound showed signs of improvement and under the influence of the anxiolytic (diazepam repeated every 8 h), the dolphin appeared settled and started to accept fish. However, radiographs had revealed the gravity and irreversibility of the lesions. On the third day in rehabilitation, blood tests showed severe leucocytosis (29,850 cells/µL). The dolphin began to vomit after feeding. As the frequency of vomiting increased over time, feeding was halted, and the dolphin was only provisioned with gavage fluids, as well as injectable antiemetics and antibiotics. On the following morning (10^th^ February 2015), the dolphin’s condition had further deteriorated and was unable to stay afloat unassisted. Blood analysis revealed a significant decrease in total white blood cell count (from ~ 30,000 to 8,000 cells/µL), as well as severe electrolyte and biochemical derangements, including hypernatraemia (187 mmol/L), hyperkalaemia (7.08 mmol/L), hyperphosphataemia (4.25 mmol/L), hypoglycaemia (2.2 mmol/L), and uraemia (38.8 mmol/L). With these added signs of irreversible multiple organ failure, it was decided to humanely euthanise WL212 through intravenous lethal injection.

### Postmortem examination

**Fig. 3:**
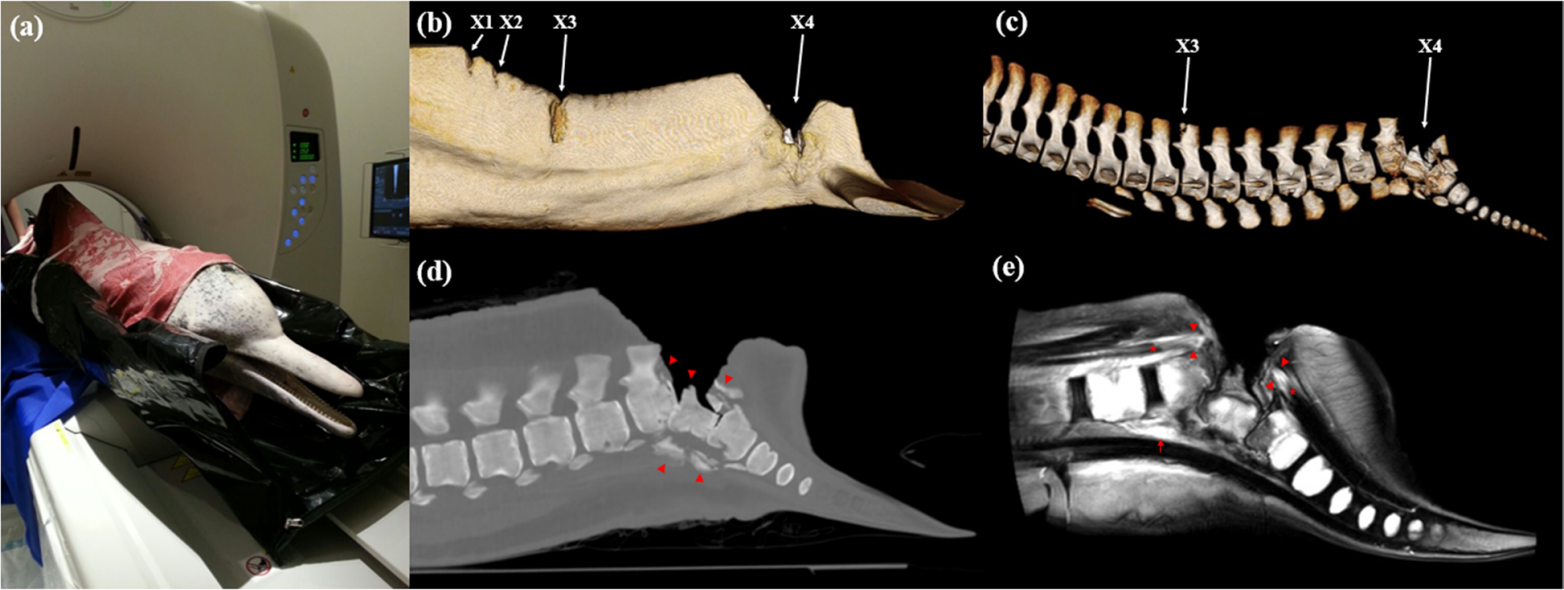
Virtopsy findings of the traumatic injuries in the severely injured *Sousa chinensis* (WL212). **a** The carcass was placed in prone position on the couch top for postmortem computed tomography (PMCT) examination. **b** Three-dimensional volume rendered image (3DVRI) from PMCT showing the lumbocaudal region of WL212 with cut chop wounds on the dorsal aspect (X1-X4), with the most severe wound at the caudal peduncle (X4). Note that parts of the dorsal fin and tail flukes were out of the scan field and thus not reconstructed. **c** 3DVRI from PMCT showing the bony structures of the caudal region, notably the fracture at the spinous process of the 4^th^ caudal vertebra at X3 and the open comminuted fracture affecting the 10^th^ to 12^th^ caudal vertebrae at X4. **d **Mid-sagittal PMCT showing the open comminuted fracture of the 10^th^ to 12^th^ caudal vertebrae at X4 (arrowheads). **e** T2-weighted postmortem magnetic resonance imaging (PMMRI) showing details of the neuromuscular injuries associated with X4, including the discontinuation of the spinal cord and spinal canal (asterisks and arrowheads) with scar tissue formation around the fractured vertebrae and spinal canal, and a hyperintense area ventral to the vertebral column suggestive of localised oedema (arrow)

Within one hour of euthanasia, the carcass of WL212 was transported to a local veterinary imaging centre for the virtopsy examination using postmortem computed tomography and magnetic resonance imaging (PMCT and PMMRI). As part of the local cetacean stranding response programme, all retrieved stranding cases undergo routine PMCT scanning prior to conventional necropsy [29]. PMCT was performed in prone position using a Toshiba 16-row multi-slice spiral CT scanner Alexion™ (Toshiba Medical Systems, Tochigi, Japan) (Fig. 3a), with the following scan parameters: 120 kV, 80 mA, 1 mm slice thickness, and scan field of view of 390 mm. Subsequently, a focal examination of the peduncle area using PMMRI was performed with a 0.25 Tesla Esaote Vet-MR Grande scanner with a standard quadrature abdominal coil (Esaote, Genoa, Italy). The following MR sequences were performed: T1-weighted sagittal (18 slices, 3.5 mm thickness, TR 524.3 ms, TE 8.0 ms, reconstruction matrix of 768 × 768, and FOV of 449 mm) and T2-weighted sagittal (18 slices, 4 mm thickness, TR 3000 ms, TE 120 ms, reconstruction matrix of 1280 × 1280, and FOV of 450 mm). Both CT and MR scans were reconstructed in the TeraRecon Aquarius iNtuition workstation (TeraRecon, San Mateo, CA) and interpreted by a board-certified radiographer and imaging researcher with experiences in cetacean virtopsy (BCWK) alongside certified veterinarians (PRM, SMC). After virtopsy, the carcass was transported back to Ocean Park Hong Kong for a gross necropsy carried out by the attending veterinarians (PRM, SMC, NF, FKL).

**Fig. 4:**
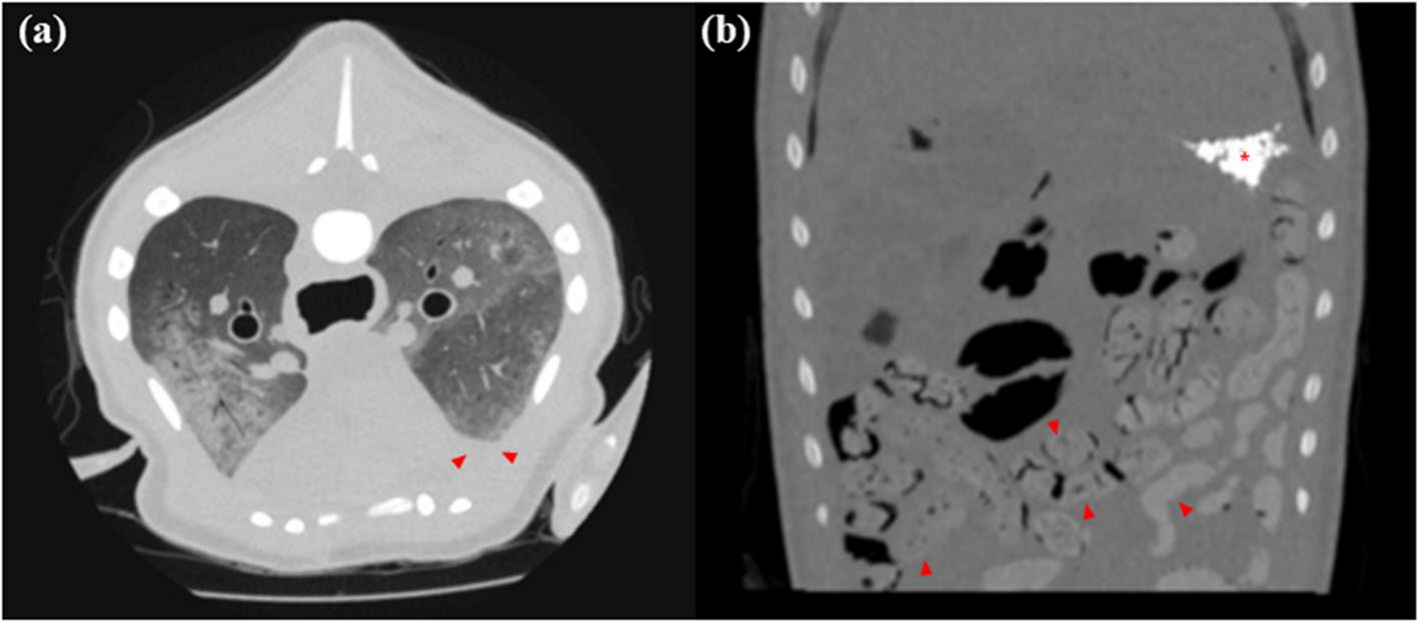
Other virtopsy findings in the severely injured *Sousa chinensis* (WL212). **a** Axial postmortem computed tomography (PMCT) at mid-thorax level showing diffused ground-glass opacification across the right lung and a focal area with more severe consolidation in the ventral left lung. There was also mild pleural effusion accumulated at the costophrenic angle shown by the obscured edge of the ventral right lung (arrowheads). **b** Coronal PMCT of the cranial-to-mid abdomen showing hyperattenuated material (~ 1300–2300 HU) in the stomach which was found to be residual fishbones (asterisk), and less hyperattenuated content (~ 180–300 HU) throughout the intestines that was compacted alongside with abnormal apperance of the intestinal lining (arrowheads)

PMCT of WL212 showed the four lacerations on the dorsal aspect from the lower lumbar region to caudal peduncle corresponding to earlier observations (Fig. 3b-d). The two wounds immediately caudal to the dorsal fin (X1 and X2) extended only into the blubber, whereas X3 severed the paraspinal muscles and fractured the spinous process of the 4^th^ caudal vertebra. The wound at the caudal peduncle (X4) penetrated the vertebral column and caused open comminuted fractures of the 10^th^ to 12^th^ caudal vertebrae. Misalignment and misangulation of the caudal peduncle region were noted with some degree of adaptation. PMMRI was used for a more detailed assessment on X4 and the adjacent areas (Fig. 3e). Discontinuation of the spinal cord with complete closure of the spinal canal, as well as the open comminuted fracture at X4 was noted on PMMRI. There was also evidence of healing with the proliferation of granulation tissues around the fractured vertebrae and the spinal canal. Moderate fluid accumulation indicative of localised oedema and inflammation was also observed in the muscles ventral to the vertebral column. Aside from the traumatic injuries, PMCT revealed focal consolidation and diffuse ground-glass opacification patterns in both lungs, especially the left lung, and the presence of fluid in the pleural cavity (Fig. 4a), suggestive of pulmonary oedema and mild pleural effusion. Hyperattenuated content was noted in the stomach and compacted faecal content was seen in the intestines (Fig. 4b), with abnormal appearance of the intestinal lining across several sections of the intestines, indicating impaction and possible enteritis.

Detailed assessment upon gross necropsy showed that the peduncle wound (X4) had exposed the three fractured vertebral bodies and was the site of deep bone infections. The exposed soft tissues appeared macerated and secondarily infected. Granulation tissues was also present around the wound. The other three cut wounds (X1, X2, X3) measured 5 cm, 4 cm, and 12 cm in depth, respectively (Fig. 1c-1d). The individual was also confirmed to be sexually mature based on the presence of spermatozoa when examined microscopically (although in rare numbers likely due to chronic injuries). Histopathological assessment of the soft tissues at the peduncle wound revealed focally extensive acute inflammation with necrosis and haemorrhage, with fibroangiomatosis peripheral to the inflammation. Concurring with PMCT, pulmonary oedema was observed in both lungs, with reddish foam seen grossly and multifocal bronchoalveolar oedema and haemorrhage diagnosed microscopically. In the peritoneal cavity, inflammation was seen on the serosal surfaces of the omentum and the cranial portion of the small intestines. Undigested fish bones were found in the stomach chambers, while the distal small intestine and the proximal large intestine were filled with dehydrated and compacted faeces. Multifocal ulcers and haemorrhages of the mucosal surface were seen throughout the stomach chambers and small intestines, especially in the fundic stomach and duodenum, where severe and extensive gastroenteritis was also diagnosed microscopically. Other findings included generalised inflammation of the spleen, kidneys, and bladder. The severe traumatic injuries and the resulting chronic septicaemia and emaciation developed over weeks in the wild, led to the health conditions justifying the humane euthanasia of WL212. The attempted rehabilitation was further compromised by gastrointestinal complications and respiratory disease.

## Discussion and conclusions

As with other wildlife, cetaceans are known to have the ability to withstand and recover from severe traumatic injuries [30]. In wild cetaceans, the prognosis for traumatic injuries varies depending on the severity and the location affected, nature and extent of the injuries, as well as the overall health status of the individual. Using case studies of injured common dolphins (*Delphinus delphis*), Olaya-Ponzone et al. [7] developed a model that categorises injuries in cetaceans and correlates the depth and severity of injuries with the chances of survival (i.e., severe injuries that penetrated bones have lower chances of survival compared to injuries that only affected the skin and blubber). It has been documented in several cetacean species that individuals were able to survive following deep soft tissue injuries from sharp force traumas caused by vessel collision. Visser and Fertl [23] documented an orca (*Orcinus orca*) that survived a sharp trauma that bisected the dorsal fin, with fully healed wounds eight months after the initial observation of injury despite the permanent disfigurement. Similar sublethal injuries caused by propeller strike was also reported in SC from Hong Kong waters [18], as well as observed by local cetacean researchers (BCWK and PRM, personal observations). In bottlenose dolphins (*Tursiops truncatus*), individuals were able to recover and reproduce after suffering from soft tissues injuries, including partial amputation of the tail fluke from vessel collision [8]. In reports where continuous monitoring of the injured cetaceans were possible, superficial injuries often healed in weeks, while deeper injuries that affected the muscles may take up to months or years to apparently heal [7, 19, 21].

For vessel collision cases, the prognosis is typically poor when involving injuries to the skeletal elements, particularly the vertebral column in cetaceans. Despite the severity, such injuries may not be immediately fatal and instead can result in extended stress, suffering, and debilitation prior to the eventual mortality, which constitute as welfare issues for the affected individuals [9, 10]. For example, Dwyer et al. [20] documented a bottlenose dolphin that suffered from multiple sharp trauma wounds on the dorsal aspect of the lumbocaudal region which appeared to extend into the vertebral column. The individual survived for at least 23 days with evidence of wound healing in granulation tissue formation but was thereafter presumed dead as it was no longer sighted with the typical social association [20]. In the current case study, WL212 was able to survive in the wild for at least 22 days post-injury under monitoring despite the severe traumatic injuries that resulted in altered mobility, septicaemia, and emaciation. Given the importance of the caudal peduncle region in the cetacean locomotion and the severity of the injuries seen during field monitoring and postmortem examination, the prognosis of WL212 without any veterinary intervention in the wild was likely fatal. The prolonged “strike-mortality interval” has negative welfare implication on animals affected by vessel-related injuries [9], and it has been suggested that humans have responsibility to mitigate vessel collision incidents and reduce welfare issues associated with vessel-related mortality and injuries [31]. In addition, although not assessed in the current study, glucocorticoid or other endocrine hormone levels measured from serum or faecal samples have been used as indicators of stress response in cetaceans [32, 33]. Differences in faecal glucocorticoid metabolites level were linked to exposure to either acute or chronic anthropogenic stressors in North Atlantic right whales (*Eubalaena glacialis*) [33]. Therefore, if similar cases are encountered in the future, stress hormone levels can potentially be used to evaluate the conditions of the animal during the course of rehabilitation, in conjunction with clinical examination and other diagnostic tests.

The routine application of virtopsy on stranded cetaceans in Hong Kong began in 2014 [29, 34], and has demonstrated to be effective in evaluating and documenting various pathologies and traumatic injuries in cetaceans [11, 35–37], especially related to vessel collision. In this case, PMCT was used to assess the skeletal fractures related to the traumatic injuries and gross organ pathologies, while PMMRI was used to evaluate the neuromuscular trauma at the peduncle wound [34, 37]. The strengths of these two imaging modalities effectively complemented each other during the postmortem investigation and produced a digital documentation of the traumatic injuries in WL212 as supplement to conventional necropsy [34, 35, 38]. More recently, three-dimensional surface scanning (3DSS) has been incorporated into the cetacean virtopsy protocol in Hong Kong to improve the ability of documenting the external features of the carcass, including wounds and lesions [39, 40]. This technique produces true-to-scale 3D models with colour and texture and is a marked improvement over the conventional method of written and photographic records used during gross necropsy, which often limited wound assessment to linear measurements or two-dimensional documentation in photographs. In addition, using appropriate image rendering techniques such as 3D volume rendered image [35, 41], CT data can also be reconstructed into 3D models that depict internal features of the carcass including soft tissue damage and fractures caused by vessel collision. By combining various techniques (PMCT, PMMRI, 3DSS), virtopsy can comprehensively assess and document the internal and external features of cetacean carcasses in a digitalised and 3D fashion, to facilitate conventional necropsy during postmortem investigations.

In human forensic medicine, the documentation of traumatic injuries with virtopsy enables the reconstruction and interpretation of the injury-causing instruments for forensic investigation [42, 43]. In stranding cases involving vessel collision, virtopsy can potentially be applied in the future to analyse the injury patterns and deduce the causative instruments, notably the propellers that produce distinctive patterns of sharp force trauma. Similar research was conducted in Florida manatees (*Trichechus manatus latirostris*), where wound characteristics such as shape, width, depth, and span between incisions were documented using photographic techniques and analysed to deduce the size and types of propellers that likely inflicted the vessel-related injuries [6]. This can allow the identification and subsequent management of high-risk vessels to mitigate vessel collision events. As cetacean virtopsy can generate accurate and calibrated 3D models that cover both internal and external aspects of cetacean carcasses, including injury patterns from vessel-related cases [35, 39], this technique may be applied in the future for accident scene reconstruction (e.g., the direction and force of impact) and identification of the propeller or vessel type involved. Overall, the application of virtopsy in cetacean stranding programme can facilitate postmortem investigation alongside conventional necropsy [29], and particularly may provide insights for the management and mitigation of vessel collision incidents in wild cetaceans.

## Data Availability

Not applicable.

## Abbreviations

3DVRI: Three-dimensional volume rendered image
PMCT: Postmortem computed tomography
PMMRI: Postmortem magnetic resonance imaging
SC: *Sousa chinensis* (Indo-Pacific humpback dolphin)
3DSS: Three-dimensional surface scanning
